# Associations of modified triglyceride-glucose indices with risks of dementia subtypes and brain structure: a prospective cohort study

**DOI:** 10.3389/fneur.2026.1750736

**Published:** 2026-02-26

**Authors:** Hanying Duan, Jiayao Liu, Xiaodong Pan, Tianwen Huang, Xiaochun Chen

**Affiliations:** Department of Neurology, Fujian Medical University Union Hospital, Fuzhou, China

**Keywords:** Alzheimer’s disease, vascular dementia, triglyceride-glucose index, TyG-BMI, TyG-WC

## Abstract

**Background:**

The associations of modified triglyceride-glucose (TyG) indices with risks of dementia subtypes and brain structural changes remain unclear. This study prospectively examines whether modified TyG indices, including TyG with body mass index (TyG-BMI) and TyG with waist circumference (TyG-WC), are associated with the risks of Alzheimer’s disease (AD) and vascular dementia (VaD) and with structural brain alterations.

**Materials and methods:**

This study analyzed 356,454 dementia-free participants from the UK Biobank. Multivariable-adjusted Cox proportional hazards models were used to estimate hazard ratios (HRs) for incident AD and VaD. Restricted cubic spline (RCS) analyses assessed nonlinear relationships. Linear regression models evaluated associations between modified TyG indices and brain structures, including hippocampal volume and white matter hyperintensity (WMH) volume. Subgroup analyses and sensitivity analyses were performed to test robustness.

**Results:**

During follow-up, 2,594 AD and 1,386 VaD cases were identified. In fully adjusted Cox models, both TyG-BMI and TyG-WC showed clear dose–response patterns with dementia risk. Using Q5 as the reference, participants in the lowest sextile (Q1) had a 47% higher risk of AD for TyG-BMI (HR = 1.47, FDR-adjusted *p* < 0.001) and a 23% higher risk for TyG-WC (HR = 1.23, FDR-adjusted *p* = 0.019), while those in the highest sextile (Q6) also tended to have increased AD risk. By contrast, VaD risk increased with higher modified TyG levels, and participants in the highest sextile had 32 and 45% higher VaD risk for TyG-BMI and TyG-WC, respectively (TyG-BMI: HR = 1.32, FDR-adjusted *p* = 0.029; TyG-WC: HR = 1.45, FDR-adjusted *p* = 0.011). Multivariable-adjusted restricted cubic spline analyses confirmed significant nonlinear relationships, showing a broad U-shaped association of modified TyG indices with AD and a J-shaped association with VaD. Higher modified TyG indices were additionally linked to larger hippocampal volume but greater WMH burden. The associations remained robust in multiple sensitivity analyses.

**Conclusion:**

Modified TyG indices show nonlinear, differential associations with AD and VaD risks, and are linked to structural brain alterations. These findings highlight the importance of metabolic health in dementia prevention and brain aging.

## Introduction

The accelerating pace of global population aging has made dementia one of the most formidable public-health challenges in the 21st century. Current epidemiological models predict a threefold increase in global dementia prevalence by mid-century if current demographic and risk factor trends persist (1). Among various dementia subtypes, Alzheimer’s disease (AD) and vascular dementia (VaD) predominate, constituting 60–80% and 10–20% of cases, respectively, (2). While AD is pathologically characterized by amyloid-*β* plaques and neurofibrillary tangles, and VaD by cerebrovascular lesions, emerging evidence suggests that metabolic dysregulation - particularly insulin resistance (IR) and obesity - represents a shared modifiable risk factor for both entities (3–6). This paradigm shift underscores the urgent need to elucidate how metabolic perturbations contribute to divergent dementia pathways through distinct pathophysiological mechanisms.

Insulin resistance, a key mediator in the crosstalk between type 2 diabetes and neurodegeneration, has garnered increasing attention as a potential therapeutic target (7). Although providing the most direct physiological measurement of insulin-mediated glucose disposal, the hyperinsulinemic-euglycemic clamp’s requirements for specialized facilities and prolonged testing protocols have accelerated the validation of simpler surrogate parameters. The triglyceride-glucose (TyG) index, integrating lipid-glucose metabolic features, has emerged as a robust IR proxy with established predictive value for cardiovascular outcomes (8, 9). Recent epidemiological studies further suggest its utility in dementia risk stratification. Korean National Health Insurance cohort revealed TyG elevation independently predicted increased risks of both AD and VaD (10), meanwhile, stratified analyses from the China Health and Retirement Longitudinal Study showed that the association between TyG and cognitive decline was more pronounced in relatively younger populations (11). These findings suggest that the metabolic-cognitive relationship may be modulated by age.

Mechanistically, neuroimaging studies provide biological plausibility for TyG-dementia associations. Findings from the Alzheimer’s Disease Neuroimaging Initiative revealed that elevated TyG combined with body mass index (TyG-BMI) was associated with reduced tau phosphorylation, increased cerebrospinal fluid (CSF) Aβ42 levels, and preserved hippocampal volume (12), suggesting a paradoxical neuroprotective role in AD pathogenesis. This observation conflicts with conventional metabolic risk paradigms but aligns with the “obesity paradox” phenomenon observed in elderly populations (13). Such discrepancies underscore the need to investigate non-linear relationships between composite metabolic indices and dementia subtypes.

Despite notable research progress, critical knowledge gaps remain. Current literature lacks systematic investigations into the predictive performance of TyG-BMI and TyG combining with waist circumference (TyG-WC) in clinical outcomes. Second, the distinct pathophysiological mechanisms underlying AD and VaD may lead to differential associations with metabolic markers, yet current studies rarely explore subtype-specific pathways. Third, the interaction between age and metabolic indices in modulating dementia risk remains insufficiently understood.

To address these gaps, we performed a large-scale prospective analysis in the UK Biobank (UKB) cohort with three primary objectives: to systematically examine the associations of modified TyG indices (TyG-BMI and TyG-WC) with the incidence of AD and VaD; to characterize non-linear dose–response relationships and subtype-specific risk patterns; to explore the impact of modified TyG indices on brain structural changes. Our findings aim to refine metabolic risk stratification frameworks and inform precision prevention strategies for dementia subtypes.

## Method

### Study population and sample

The UK Biobank study is an ongoing population-based prospective cohort that enables comprehensive investigation of multifactorial disease etiology, incorporating genomic, environmental, and behavioral determinants (14). Between 2006 and 2010, approximately 500,000 participants were recruited across the United Kingdom. Comprehensive baseline data were collected, including questionnaire information, physical measurements, biological samples (blood, urine, and saliva), and imaging assessments (e.g., brain MRI scans). In addition, the UKB has established long-term follow-up through linkage with National Health Service health records, including hospital admissions, outpatient visits, cancer registries, and death registries (15). The Northwest Multi-centre Research Ethics Committee approved the UK Biobank study protocol, and written informed consent was obtained from all participants at baseline. The application number for the present study is 470,494.

**Figure 1:**
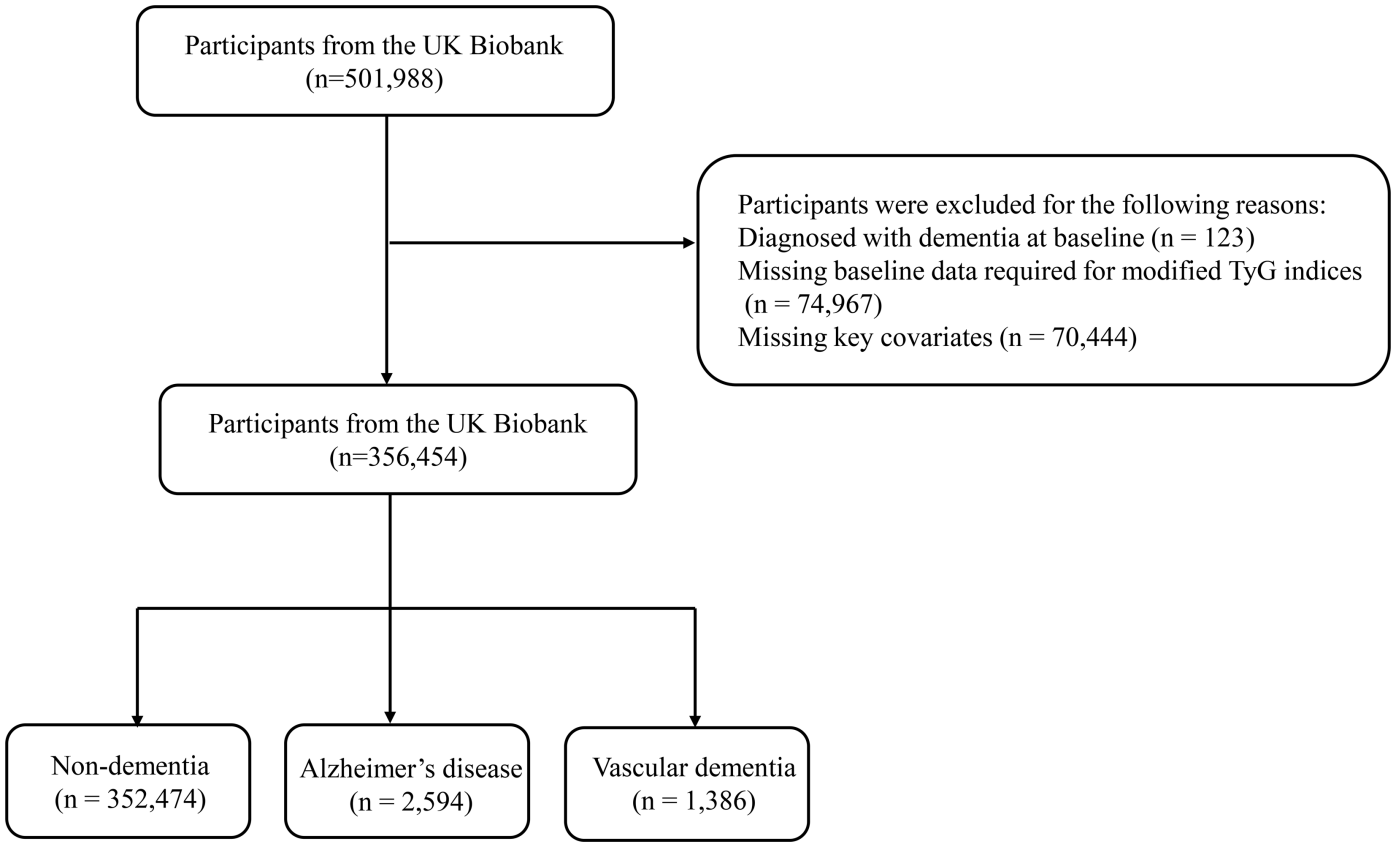
Flowchart illustrating the selection process of study participants.

In this study, we initially screened a total of 501,988 participants from the UKB. We first excluded 123 individuals who had been diagnosed with dementia at baseline. Subsequently, 74,967 participants were excluded due to missing data on the modified TyG indices (including blood glucose, triglyceride (TG), BMI, and WC). An additional 70,444 individuals were excluded due to missing key covariate information on age, sex, apolipoprotein E (APOE) genotype, or ethnicity. Ultimately, 356,454 participants met the inclusion criteria and were included in the final analyses (Figure 1).

### Definitions of modified TyG indices

The modified TyG indices, including TyG-BMI and TyG-WC, are extensions of the traditional TyG index, incorporating BMI and WC into their respective calculations. The traditional TyG index is derived from fasting blood glucose (FBG) and TG levels measured at baseline. The formulas are as follows: TyG index = log [TG (mg/dL) × FBG (mg/dL)/2]; TyG-BMI index = TyG index × BMI; TyG-WC index = TyG index × WC.

### Determination of dementia onset

The primary outcomes of this study are AD and VaD. The determination of dementia types is based on multiple data sources, including hospitalization and diagnostic records from England’s hospital incidence statistics, Scotland’s incidence records, and Wales’ patient database. Additionally, death registration data was collected from the National Health Service digital records in England and Wales, as well as from the Information Services Division of Scotland, to identify more cases. The classification of dementia types was based on both the International Classification of Diseases Ninth Revision (ICD-9) and Tenth Revision (ICD-10) codes. AD was defined by ICD-10 codes F00 and G30, and ICD-9 code 331.0. VaD was defined by ICD-10 codes F01 and I67.3, and ICD-9 code 290.4.

### Assessment of covariates

The covariates included in this study were: age, sex, ethnicity, APOE genotype, educational level, Townsend Deprivation Index (TDI), household income, smoking status, alcohol consumption, dietary habits, sleep patterns, hypertension, diabetes, dyslipidemia, history of stroke, history of atrial fibrillation (AF), and the use of glucose-lowering and lipid-lowering medications. For imaging outcomes, we also adjusted for total brain volume.

Race was dichotomized (White/non-White). Smoking and alcohol use were categorized as current, former, or never users (16). Diet quality was assessed using a dietary score based on seven components (vegetables, fruits, fish, whole grains, refined grains, processed meats, and unprocessed meats), with a total score ranging from 0 to 7; higher scores indicated greater adherence to healthy dietary guidelines (17, 18). Sleep patterns were evaluated using a composite sleep score derived from five factors: chronotype, sleep duration, insomnia, snoring, and daytime sleepiness. The total score ranged from 0 to 5 and was used to categorize sleep patterns as healthy (4–5), intermediate (2–3), or poor (0–1) (19).

### Structural MRI measurement analysis of the brain

The MRI imaging protocol used in this study was designed by the UK Biobank Imaging Working Group, and the detailed image acquisition protocol and data processing methods have been thoroughly described in previously published literature (20). All neuroimaging data were standardized and acquired using the Siemens Skyra 3 T MRI scanner, and image segmentation and quantification were performed based on the automated processing workflow established by the UKB. This study focused on three key neuroimaging measures: total brain volume, white matter hyperintensity (WMH) volume, and hippocampal volume.

### Statistical analysis

This study, based on data from the UKB, systematically evaluates the association between modified TyG indices and dementia risk, as well as its impact on brain structure. Descriptive statistics present categorical variables as numbers (%) and continuous variables as means (standard deviations (SD)). Between-group differences were evaluated using chi-square tests for proportions and ANOVA for continuous measures. Missing covariate data were addressed using multiple imputation by chained equations (MICE) to minimize potential bias.

To quantify the relationship between modified TyG indices and dementia risk, we constructed a series of Cox proportional hazards models with multiple levels of adjustment: Model 1 adjusted for age, sex, ethnicity, APOE genotype, educational level; Model 2 further adjusted for TDI, household income, smoking, alcohol consumption, diet, and sleep patterns; Model 3 additionally included hypertension, diabetes, dyslipidemia, history of stroke, history of atrial fibrillation (AF), and the use of glucose-lowering and lipid-lowering medications.

To reveal the nonlinear relationship between modified TyG indices and dementia risk, we used a restricted cubic spline (RCS) model (4 knots) to fit the dose–response curve, adjusting for the covariates in Model 3, and evaluated the statistical significance of the nonlinear trend using likelihood ratio tests. For the cross-sectional associations between modified TyG indices and brain structures, we used generalized linear regression to calculate the standardized *β* coefficient (reflecting the change in brain volume per 1 SD increase in modified TyG indices) and its 95% CI.

In the subgroup analysis, we stratified participants by age, sex, ethnicity, APOE ε4 carrier status, hypertension, hyperlipidemia, diabetes, and fasting duration, and evaluated the interactions between these subgroup variables and the modified TyG indices using likelihood ratio tests. Two sensitivity analyses were conducted to verify result robustness: (1) excluding those who developed dementia during the initial 2 years of follow-up to address potential reverse causality bias; (2) using the original TyG index to replace modified TyG indices and re-evaluating its association with dementia to assess whether the relationship between modified TyG indices and dementia is stable.

All analyses were conducted using R (v4.4.2) with the “mice,” “survival,” and “rms” packages. A two-sided *p* value < 0.05 was considered statistically significant. To account for multiple comparisons, false discovery rate (FDR)-adjusted *p* values were calculated using the Benjamini-Hochberg procedure.

## Results

### Basic characteristics of participants

Table 1 describes the baseline characteristics of 356,454 participants, including 352,474 non-dementia controls and 3,980 dementia cases (2,594 AD, 1,386 VaD). The overall population had a mean age of 56.50 ± 8.11 years, 54.33% female, 94.09% white, and a mean follow-up of 13.27 ± 2.05 years.

**Table 1:** Baseline characteristics of study participants by dementia status.

Characteristic	Overall	Non-dementia^1^	AD^1^	VaD^1^	*P*-value^2^
Sample size	356,454	352,474	2,594	1,386	
Follow time, years (mean (SD))	13.27 (2.05)	13.31 (2.01)	10.22 (2.94)	10.12 (2.94)	<0.001*
Age, years (mean (SD))	56.50 (8.11)	56.41 (8.10)	64.70 (4.20)	64.82 (4.09)	<0.001*
Sex = Female (%)	193,664 (54.33%)	191,698 (54.39%)	1,388 (53.51%)	578 (41.70%)	<0.001*
Ethnicity = White (%)	335,371 (94.09%)	331,564 (94.07%)	2,486 (95.84%)	1,321 (95.31%)	<0.001*
APOE ε4 carriers (%)	101,830 (28.57%)	99,529 (28.24%)	1,616 (62.30%)	685 (49.42%)	<0.001*
Education = College (%)	117,378 (32.93%)	137,031 (32.46%)	539 (20.78%)	235 (16.96%)	<0.001*
TDI (mean (SD))	−1.35 (3.06)	−1.36 (3.06)	−1.23 (3.17)	−0.80 (3.41)	<0.001*
TyG (mean (SD))	8.71 (0.57)	8.71 (0.57)	8.76 (0.55)	8.86 (0.62)	<0.001*
TyG-BMI (mean (SD))	239.32 (49.38)	239.26 (49.36)	242.00 (47.57)	254.19 (54.18)	<0.001*
TyG-WC (mean (SD))	788.10 (146.88)	787.76 (146.81)	800.83 (142.30)	848.54 (157.60)	<0.001*
Healthy diet score (mean (SD))	2.81 (1.29)	2.97 (1.28)	2.81 (1.29)	2.85 (1.29)	<0.001*
Smoking status = Never (%)	196,954 (55.25%)	195,076 (55.34%)	1,292 (49.81%)	586 (42.28%)	<0.001*
Drinking status = Never (%)	15,918 (4.47%)	15,633 (4.44%)	192 (7.40%)	93 (6.71%)	<0.001*
Sleep pattern = Healthy (%)	192,157 (53.91%)	190,143 (53.95%)	1,360 (52.43%)	654 (47.19%)	<0.001*
Hypertension history = Yes (%)	81,134 (22.76%)	79,868 (22.66%)	776 (29.92%)	490 (35.35%)	<0.001*
Diabetes history = Yes (%)	4,750 (1.33%)	4,635 (1.31%)	65 (2.51%)	50 (3.61%)	<0.001*
Dyslipidemia history = Yes (%)	8,661 (2.43%)	8,545 (2.42%)	78 (3.01%)	38 (2.74%)	0.12
Stroke history = Yes (%)	3,900 (1.09%)	3,759 (1.07%)	53 (2.04%)	88 (6.35%)	<0.001*
AF history = Yes (%)	743 (0.21%)	732 (0.21%)	8 (0.31%)	3 (0.22%)	0.5
Glucose-lowering medication = Yes (%)	3,550 (1.00%)	3,482.00 (0.99%)	33.00 (1.27%)	35.00 (2.53%)	<0.001*
Lipid-lowering medication = Yes (%)	62 (0.02%)	56.00 (0.02%)	1.00 (0.04%)	5.00 (0.36%)	<0.001*

^1^n (%); ^2^One-way analysis of means (not assuming equal variances); Pearson’s Chi-squared test. * Indicates statistical significance. AD, Alzheimer’s disease; VaD, vascular dementia; APOE, apolipoprotein E; TDI, Townsend Deprivation Index; TyG, triglyceride-glucose index; BMI, body mass index; WC, waist circumference; TyG-BMI, TyG combining with body mass index; TyG-WC, TyG combining with waist circumference; AF, atrial fibrillation; SD, standard deviation.

Compared to non-dementia controls, dementia groups were older, had fewer females, higher white ethnicity, and greater socioeconomic disadvantages (lower college education rates and higher deprivation scores). Genetically, dementia cases—particularly those with AD—had a markedly higher prevalence of APOE ε4 carriers. Unhealthy lifestyle factors were also more common among dementia cases, including lower healthy diet scores, higher rates of smoking and less favorable sleep patterns (*p* < 0.001). Medically, histories of hypertension, diabetes, and stroke were significantly more prevalent in both dementia subtypes (*p* < 0.001), while dyslipidemia and AF histories did not differ significantly across groups (*p* = 0.12 and 0.5, respectively). Use of glucose- and lipid-lowering medications was also higher among dementia groups.

### Associations between modified TyG indices and dementia outcomes

In Cox proportional hazards models, both TyG-BMI and TyG-WC were independently associated with AD and VaD risk after full adjustment (Table 2). In the Model 3, a 1-unit increment in TyG-BMI was linked to 0.17% decrease in AD risk (HR = 0.9983, FDR-adjusted *p* = 0.001) and a 0.3% increase in VaD risk (HR = 1.003, FDR-adjusted *p* < 0.001). Similarly, each 1-unit increase in TyG-WC predicted a 0.04% reduction in AD risk (HR = 0.9996, FDR-adjusted *p* = 0.042) and a 0.12% increase in VaD risk (HR = 1.0012, FDR-adjusted *p* < 0.001). The sextile-based analysis revealed a clear dose–response relationship. In the fully adjusted model using Q5 as the reference, both TyG-BMI and TyG-WC exhibited a significant U-shaped association with the risk of AD. Participants in the lowest sextile (Q1) had a significantly increased risk of AD (TyG-BMI: HR = 1.47, FDR-adjusted *p* < 0.001; TyG-WC: HR = 1.23, FDR-adjusted *p* = 0.019), while those in the highest sextile (Q6) also showed a trend toward increased risk, although the associations were not statistically significant (TyG-BMI: HR = 1.1, FDR-adjusted *p* = 0.177; TyG-WC: HR = 1.07, FDR-adjusted *p* = 0.309). In contrast, the risk of VaD showed a monotonically increasing pattern, with participants in the highest sextile (Q6) having significantly elevated risks (TyG-BMI: HR = 1.32, FDR-adjusted *p* = 0.029; TyG-WC: HR = 1.45, FDR-adjusted *p* = 0.011) (Table 2).

**Table 2:** The HR (95% CI) of dementia according to modified TyG indices in the three models.

Categories	Model 1			Model 2			Model 3		
	HR (95% CI)	*P-*value	Adjusted *P*	HR (95% CI)	*P-*value	Adjusted *P*	HR (95% CI)	*P-*value	Adjusted *P*
AD									
TyG-BMI									
Continuous variable per unit	0.999 (0.9982, 0.9999)	0.022*	0.078	0.9985 (0.9976, 0.9993)	<0.001*	0.002*	0.9983 (0.9975, 0.9992)	<0.001*	0.001*
Sextile									
Q1	1.42 (1.24, 1.62)	<0.001*	<0.001*	1.46 (1.27, 1.66)	<0.001*	<0.001*	1.47 (1.28, 1.68)	<0.001*	<0.001*
Q2	1.1 (0.97, 1.26)	0.14	0.229	1.14 (1, 1.3)	0.052	0.094	1.15 (1.01, 1.31)	0.036*	0.072
Q3	1.13 (1, 1.28)	0.055	0.121	1.16 (1.03, 1.32)	0.018*	0.047*	1.17 (1.03, 1.33)	0.013*	0.033*
Q4	1.11 (0.98, 1.26)	0.09	0.162	1.13 (1, 1.28)	0.051	0.094	1.14 (1.01, 1.29)	0.041*	0.074
Q5	Reference			Reference			Reference		
Q6	1.17 (1.03, 1.32)	0.017*	0.078	1.12 (0.98, 1.27)	0.089	0.134	1.1 (0.97, 1.25)	0.128	0.177
TyG-WC									
Continuous variable per unit	0.9999 (0.9996, 1.0002)	0.431	0.518	0.9997 (0.9994, 1)	0.044*	0.094	0.9996 (0.9993, 0.9999)	0.019*	0.042*
Sextile									
Q1	1.17 (1.01, 1.35)	0.035*	0.09	1.22 (1.05, 1.41)	0.008*	0.024*	1.23 (1.06, 1.42)	0.006*	0.019*
Q2	1.16 (1.02, 1.32)	0.026*	0.078	1.19 (1.05, 1.36)	0.008*	0.024*	1.2 (1.05, 1.37)	0.006*	0.019*
Q3	1.04 (0.91, 1.18)	0.56	0.63	1.07 (0.94, 1.21)	0.311	0.350	1.07 (0.95, 1.22)	0.272	0.309
Q4	1.05 (0.93, 1.19)	0.418	0.518	1.07 (0.95, 1.21)	0.278	0.333	1.08 (0.95, 1.22)	0.249	0.309
Q5	Reference			Reference			Reference		
Q6	1.12 (0.99, 1.27)	0.06	0.121	1.09 (0.96, 1.23)	0.184	0.237	1.07 (0.95, 1.21)	0.275	0.309
VaD									
TyG-BMI									
Continuous variable per unit	1.0047 (1.0036, 1.0057)	<0.001*	<0.001*	1.0038 (1.0027, 1.0049)	<0.001*	<0.001*	1.003 (1.0019, 1.0041)	<0.001*	<0.001*
Sextile									
Q1	Reference			Reference			Reference		
Q2	0.83 (0.66, 1.05)	0.116	0.262	0.85 (0.68, 1.07)	0.168	0.324	0.85 (0.68, 1.06)	0.153	0.400
Q3	1.03 (0.83, 1.27)	0.781	0.827	1.05 (0.85, 1.3)	0.652	0.691	1.03 (0.83, 1.27)	0.815	0.979
Q4	0.95 (0.77, 1.17)	0.606	0.728	0.94 (0.76, 1.17)	0.600	0.675	0.91 (0.73, 1.12)	0.378	0.619
Q5	1.09 (0.89, 1.34)	0.405	0.521	1.06 (0.86, 1.31)	0.570	0.675	0.99 (0.8, 1.22)	0.931	0.980
Q6	1.59 (1.3, 1.94)	<0.001*	<0.001*	1.46 (1.19, 1.78)	<0.001*	0.001	1.32 (1.08, 1.62)	0.008*	0.029*
TyG-WC									
Continuous variable per unit	1.0018 (1.0014, 1.0022)	<0.001*	<0.001*	1.0014 (1.001, 1.0018)	<0.001*	<0.001*	1.0012 (1.0007, 1.0016)	<0.001*	<0.001*
Sextile									
Q1	Reference			Reference			Reference		
Q2	1.15 (0.9, 1.46)	0.273	0.446	1.12 (0.88, 1.43)	0.345	0.564	1.1 (0.86, 1.41)	0.432	0.649
Q3	1.12 (0.88, 1.42)	0.349	0.506	1.09 (0.86, 1.39)	0.481	0.675	1.05 (0.83, 1.34)	0.666	0.857
Q4	1.28 (1.01, 1.62)	0.039*	0.101	1.23 (0.97, 1.55)	0.089	0.266	1.17 (0.92, 1.48)	0.204	0.406
Q5	1.16 (0.91, 1.47)	0.225	0.414	1.08 (0.85, 1.37)	0.542	0.675	1 (0.79, 1.28)	0.980	0.980
Q6	1.86 (1.48, 2.34)	<0.001*	<0.001*	1.63 (1.3, 2.06)	<0.001*	<0.001*	1.45 (1.15, 1.84)	0.002*	0.011*

* Indicates statistical significance. Model 1 was adjusted for age, gender, ethnicity, education, and APOE genotype. Model 2 was further adjusted for Townsend deprivation index (TDI), smoking status, drinking status, household income, dietary habits, and sleep patterns. Model 3 was additionally adjusted for a history of hypertension, hyperlipidemia, diabetes, atrial fibrillation, stroke, and the use of glucose-lowering and lipid-lowering medications. TyG, triglyceride-glucose index; BMI, body mass index; WC, waist circumference; TyG-BMI, TyG combining with body mass index; TyG-WC, TyG combining with waist circumference; AD, Alzheimer’s disease; VaD, vascular dementia; HR, hazard ratios; CI, confidence interval.

**Figure 2:**
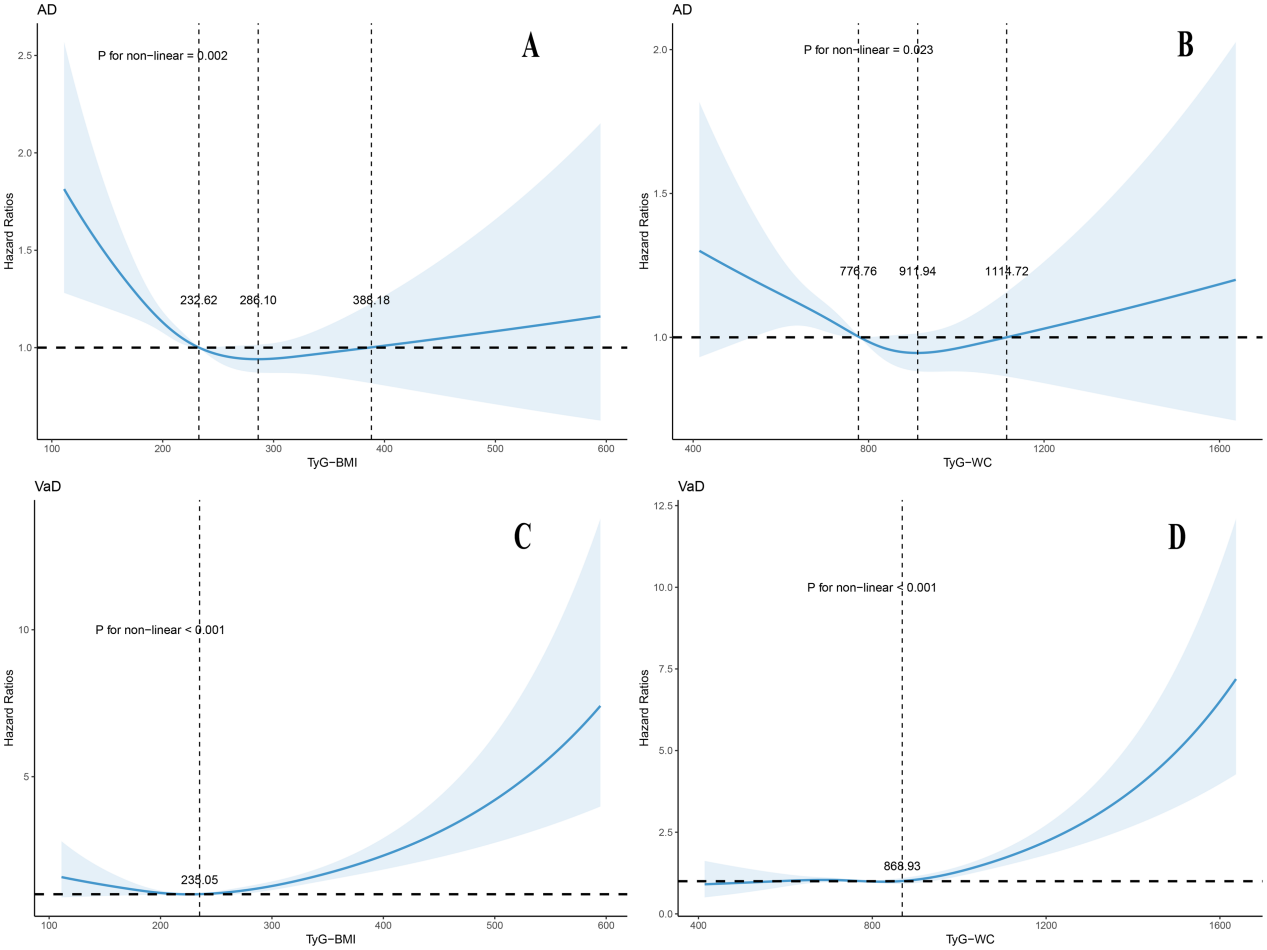
Association of modified TyG indices and dementia risk using a multivariable-adjusted restricted cubic spines model. Restricted cubic spline analysis has four knots at the 5th, 35th, 65th, and 95th percentiles of modified TyG indices. The model adjusted for age, gender, ethnicity, education, and APOE genotype, Townsend deprivation index (TDI), smoking status, drinking status, household income, dietary habits, and sleep patterns, the history of hypertension, hyperlipidemia, diabetes, atrial fibrillation, stroke, the use of glucose-lowering and lipid-lowering medications. **(A,B)** For participants with AD; **(C,D)** for participants with VaD; TyG, triglyceride-glucose index; BMI, body mass index; WC, waist circumference; TyG-BMI, TyG combining with body mass index; TyG-WC, TyG combining with waist circumference; AD, Alzheimer’s disease; VaD, vascular dementia.

The multivariable-adjusted RCS analysis revealed significant nonlinear dose–response relationships between TyG-BMI, TyG-WC, and the risks of AD and VaD (Figure 2). For AD, both indices displayed a broad U-shaped relationship. The nadir of risk occurred at TyG-BMI = 286.10, with the protective range spanning 232.62–388.18. TyG-WC showed protective effects within the range of 776.76 to 1114.72, with the lowest risk at 911.94 (P for nonlinear = 0.023). Risk gradually increased outside of these optimal ranges. In contrast, the association with VaD followed a J-shaped pattern: risk remained stable within the normal range, but increased significantly when TyG-BMI exceeded 235.05 (P for nonlinear < 0.001) or TyG-WC exceeded 868.93 (P for nonlinear < 0.001).

### Associations between modified TyG indices and brain structures

Based on the above results, we further investigated relationships between modified TyG indices and region-specific brain volumetric changes using structural MRI. After further adjusting for total brain volume on the basis of Model 3, multiple linear regression analysis revealed that both TyG-BMI and TyG-WC were independently associated with brain structural alterations. As shown in Table 3, higher levels of TyG-BMI and TyG-WC were significantly associated with increased hippocampal volume (TyG-BMI: *β* = 0.64, FDR-adjusted *p* < 0.001; TyG-WC: *β* = 0.23, FDR-adjusted *p* < 0.001), suggesting that elevated modified TyG indices may offer protective effects against hippocampal atrophy, a hallmark of AD.

**Table 3:** Associations between brain structures and modified TyG indices.

Categories	Volume of hippocampus (*n* = 45,834)			Volume of white matter hyperintensities (*n* = 44,089)		
	*β* (95%CI)	*P-*value	Adjusted *P*	β (95%CI)	*P-*value	Adjusted *P*
TyG-BMI	0.64 (0.48, 0.80)	<0.001*	<0.001*	8.85 (7.37, 10.33)	<0.001*	<0.001*
TyG-WC	0.23 (0.17, 0.28)	<0.001*	<0.001*	3.22 (2.68, 3.76)	<0.001*	<0.001*

* Indicates statistical significance. Model adjusted for age, gender, ethnicity, education, and APOE genotype, Townsend deprivation index (TDI), smoking status, drinking status, household income, dietary habits, and sleep patterns, the history of hypertension, hyperlipidemia, diabetes, atrial fibrillation, stroke, the use of glucose-lowering medications, lipid-lowering medications and total brain volume. n: number of participants; CI, confidence interval; TyG, triglyceride-glucose index; BMI, body mass index; WC, waist circumference; TyG-BMI, TyG combining with body mass index; TyG-WC, TyG combining with waist circumference.

Meanwhile, both TyG-BMI and TyG-WC were also significantly associated with greater WMH volume (TyG-BMI: *β* = 8.85, FDR-adjusted *p* < 0.001; TyG-WC: *β* = 3.22, FDR-adjusted *p* < 0.001), indicating that higher modified TyG indices are closely linked to increased small vessel disease burden, which might play a pivotal role in the development and progression of VaD.

Owing to the limited number of dementia subtype events, formal survival-based mediation analysis was not feasible in the present study. To further explore whether modified TyG indices may influence cognitive outcomes through brain structural alterations, disease-relevant brain structural measures were additionally included as covariates in the multivariable models. After further adjustment for hippocampal volume in the analyses of AD or WMH volume in the analyses of VaD, the associations between modified TyG indices and dementia risk were substantially attenuated and no longer statistically significant (Supplementary Table 1). These findings suggest that brain structural alterations may partly account for the association between modified TyG indices and dementia outcomes.

### Subgroup analyses

We performed stratified analyses to assess whether the associations between modified TyG indices and dementia risk varied across different subgroups (Supplementary Table 2). Significant interactions were found with age (TyG-BMI: *p* = 0.003; TyG-WC: *p* < 0.001) and APOE genotype (TyG-BMI: *p* = 0.003; TyG-WC: *p* < 0.001) for AD. In participants aged ≥65 years, both TyG-BMI and TyG-WC were significantly and inversely associated with AD risk, whereas no such associations were found in those under 65 years. Similarly, among APOE ε4 non-carriers, higher modified TyG indices were associated with lower AD risk, but this association was absent in ε4 carriers.

In addition, significant interactions were observed between TyG-BMI and hypertension (*p* = 0.019), TyG-BMI and hyperlipidemia (p = 0.019), and between TyG-WC and diabetes (*p* = 0.007), indicating stronger associations in individuals with these comorbidities.

For VaD, both TyG-BMI and TyG-WC showed positive associations with VaD risk, and interaction effects were observed with age (TyG-BMI: *p* < 0.035; TyG-WC: *p* < 0.001) and APOE genotype (TyG-BMI: *p* = 0.005; TyG-WC: *p* < 0.001). These associations were more prominent among younger individuals and APOE ε4 carriers.

No statistically significant interactions were detected for sex, ethnicity, or fasting duration in either AD or VaD models (all P for interaction > 0.05), suggesting that the observed associations were largely consistent across these subgroups.

### Sensitivity analyses

To assess result robustness, we conducted several sensitivity analyses. First, we excluded participants who developed dementia within the first 2 years of baseline assessment, and the associations between TyG-BMI, TyG-WC, and dementia risk remained largely unchanged, further supporting the reliability of our results (Supplementary Table 3). Second, we re-evaluated the association between the original TyG index (without incorporating BMI or WC) and the risk of dementia (Supplementary Table 4). In the continuous variable model, each one-unit increase in TyG was associated with a 12% reduced risk of AD (HR = 0.88, 95% CI: 0.82–0.94, FDR-adjusted *p* = 0.001), but an 14% increased risk of VaD (HR = 1.14, 95% CI: 1.03–1.26, FDR-adjusted *p* = 0.029). RCS analysis revealed a U-shaped association between TyG and AD risk (P for nonlinear = 0.011) and a J-shaped association with VaD risk (P for nonlinear < 0.001) (see Supplementary Figure 1). In the sextile-based model, elevated AD risk was primarily observed in the lowest TyG sextile (Q1; HR = 1.3, FDR-adjusted *p* = 0.001), while the highest sextile (Q6) showed a trend toward increased risk, though not statistically significant (HR = 1.02, FDR-adjusted *p* = 0.772). For VaD, this association was attenuated and no longer significant in Model 3 (HR = 1.13, FDR-adjusted *p* = 0.406). Taken together, these findings suggest that while the original TyG index may offer some predictive value for AD and VaD, its associations are relatively unstable. In contrast, the composite indices incorporating BMI or WC demonstrated more robust and consistent associations across analyses, indicating superior predictive performance.

## Discussion

In this large-scale prospective study based on the UKB, we systematically evaluated the associations between modified TyG indices (TyG-BMI and TyG-WC), dementia subtypes, and brain structural changes. We found a U-shaped relationship between TyG-BMI/TyG-WC and the risk of AD, indicating that both low and elevated levels were linked to increased AD risk. In contrast, a J-shaped association was observed with VaD, where only higher levels were significantly linked to elevated VaD risk. These relationships remained robust following thorough adjustment for multiple potential confounders and were more pronounced in specific subgroups. Notably, brain structural analyses revealed that higher TyG-BMI and TyG-WC levels were positively associated with greater WMH volume and larger hippocampal volume, suggesting that metabolic dysregulation may influence dementia risk through structural brain alterations. Overall, this study is the first to systematically assess the relationships between TyG-BMI, TyG-WC, and both dementia subtypes and brain structure. Our findings highlight the potential mechanistic role of insulin resistance and obesity- associated indices in the pathogenesis of different forms of dementia via brain structural changes.

The modified TyG indices (TyG-BMI, TyG-WC) synergistically capture lipid metabolism dysregulation and adiposity patterns, serving as both robust proxies for insulin resistance and holistic metabolic health biomarkers in epidemiological research. Compared to the standalone TyG index, this modified approach offers a more thorough risk evaluation. In fact, existing research on the relationship between modified TyG indices and AD is limited. A study from Tianjin Medical University’s self-established dementia cohort found a non-linear U-shaped relationship between the TyG-WC value and cognitive function, but this study did not differentiate between specific dementia subtypes (21). Our research also shows a U-shaped association between the modified TyG indices and AD, which may reflect the dual impact of insulin resistance and obesity on AD risk. Specifically, both extremely high and low levels of insulin resistance and obesity may be associated with AD risk through different mechanisms. Extremely low metabolic levels may indicate that the body is in a state of malnutrition or depleted metabolic reserves, which is closely related to frailty syndrome in the elderly. Notably, recent research has established that frailty is an independent risk factor for AD (22–25). In contrast, in high metabolic load states, abnormal insulin signaling leads to reduced synaptic plasticity, affecting tau phosphorylation, promoting amyloid-beta deposition, and inducing neuronal apoptosis, all of which have been established as core pathological mechanisms of AD (26, 27). Furthermore, insulin resistance has been shown to be closely associated with increased blood–brain barrier permeability, which may further accelerate the process of neurodegenerative changes (28). It is also noteworthy that our study found a slower rate of hippocampal volume atrophy associated with the modified TyG indices. These results align with the research by Zihao Zhang et al., further supporting the relationship between metabolic health and brain structure (12). This suggests that metabolic abnormalities may influence hippocampal atrophy, thereby affecting cognitive function.

The positive correlation between modified TyG indices and VaD risk further validates the known mechanisms of IR in cerebrovascular diseases. The TyG-BMI and TyG-WC indices collectively capture three key metabolic disturbances—hyperglycemia, dyslipidemia, and central adiposity—which synergistically promote endothelial dysfunction, accelerate atherosclerosis, and sustain pro-inflammatory states implicated in neurodegeneration (29–31). These factors accelerate the occurrence of small vessel lesions and white matter damage, ultimately promoting the development of VaD. The relationship between metabolic disorders and small vessel damage may involve several pathogenic mechanisms. First, insulin resistance leads to endothelial dysfunction, reducing nitric oxide (NO) synthesis, thereby impairing vascular dilation and promoting atherosclerosis (32, 33). Second, impaired vascular function affects cerebral blood supply, reducing oxygen and nutrient delivery to the brain, lowering cerebral glucose metabolism, and increasing the risk of thrombosis, which accelerates cerebrovascular changes (34, 35). Third, metabolic disorders affect insulin signaling and sensitivity, enhancing chronic systemic inflammation, increasing inflammatory markers (such as TNF-*α*, IL-6, and CRP), and elevating free fatty acid levels, which promote foam cell formation, thereby accelerating atherosclerosis and small vessel damage in the brain (33, 36–38). In addition, metabolic abnormalities can also affect the metabolism of factors such as cyclic guanosine monophosphate (cGMP), insulin-like growth factor-1 (IGF-1), and insulin-like growth factor-2 (IGF-2), increasing platelet aggregation and activation, which predisposes to thrombosis and ultimately leads to vascular occlusion (39–41). Finally, insulin resistance and obesity promote small vessel damage through multiple mechanisms, resulting in increased white matter hyperintensity, indicative of cerebrovascular microdamage, which ultimately leads to the development of VaD. This finding is consistent with our structural brain analysis, which shows that higher levels of TyG-BMI and TyG-WC are significantly associated with increased white matter hyperintensity volume. A 9-year prospective cohort study at Radboud University demonstrated that elevated baseline waist circumference predicted greater WMH volume progression (42). Similarly, Zhenjie Teng found through mediation analysis that the burden of severe small vessel disease mediates the relationship between higher TyG index levels and cognitive impairment, further supporting the possible mechanism by which metabolic disorders increase VaD risk through small vessel damage (43).

Notably, our stratified analysis revealed significant age-dependent differences. Among individuals aged ≥65 years, those in the lowest quintile (Q1) of TyG-BMI and TyG-WC had a significantly higher risk of AD compared to those in the middle quintile (Q5). This finding suggests that lower metabolic levels in older adults may also be detrimental, potentially reflecting the adverse effects of malnutrition or reduced metabolic reserves on neurodegenerative processes. Conversely, the association with VaD was more prominent in the <65 age group, particularly among individuals in the higher TyG-WC quintiles (Q4–Q6), where a significantly elevated risk of VaD was observed. These findings suggest that insulin resistance and metabolic dysfunction during midlife may exert stronger vascular damage, thereby promoting the development of VaD. This finding may support the pathophysiological features of the “obesity paradox”—wherein midlife obesity confers dementia risk while late-life obesity shows neutral or even protective associations. Existing evidence is consistent with this finding. A cohort study of post-stroke cognitive impairment (PSCI) revealed age-dependent associations: general obesity significantly increased PSCI risk in middle-aged patients (HR = 1.32), but showed no association in older adults (HR = 0.97) (44). Furthermore, another study found that weight loss in elderly patients predicted cognitive decline, with weight loss in later life (regardless of BMI) leading to negative health outcomes, including accelerated cognitive decline (45). The paradoxical nature of this phenomenon may be explained by several mechanisms. First, adipose tissue secretes neuroprotective peptides such as leptin (with serum leptin levels higher in the elderly than in middle-aged individuals), which can enhance hippocampal synaptic plasticity and improve cognitive function. This age-dependent endocrine regulation may explain the attenuation of cognitive impairment risk in elderly obese individuals (46, 47). Additionally, the survival effect suggests that middle-aged obese individuals with visceral fat accumulation may die prematurely, leaving behind a low-risk obesity phenotype in the elderly (48). Finally, individuals with cognitive decline may neglect health-related behaviors (such as exercise and dietary management), leading to weight loss or low body weight. In such cases, cognitive decline may be a precursor to low body weight rather than a consequence. A meta-analysis found a negative correlation between abdominal obesity and the risk of late-onset AD (HR = 0.84), with researchers suggesting that this inverse relationship may be due to confounding bias caused by involuntary weight loss during the preclinical phase of dementia (13). Thus, additional research is required to elucidate the specific mechanisms and potential causal relationships of the obesity paradox in the elderly, in order to provide more precise guidance for establishing age-stratified intervention strategies.

This study has several limitations. First, while an association was observed between the modified TyG indices and dementia subtypes, the observational design of this study—coupled with the absence of randomized controlled trials—prevents inference of causal relationships between the modified TyG indices and dementia risk. Second, owing to the limited number of dementia subtype events, formal survival-based mediation analysis could not be conducted in this study. Third, while the UKB provides a large sample, the participants are predominantly of European descent, which may limit the external validity of the findings and prevent their full generalization to other racial or regional populations. Finally, despite adjusting for multiple variables, there may still be unmeasured potential confounders that could affect the results. Therefore, future studies should validate these findings in larger, more diverse populations and elucidate underlying mechanisms through experimental models.

## Conclusion

This study reveals a complex nonlinear relationship between modified TyG indices and the risk of different dementia subtypes. Additional brain structural analyses suggest that the modified TyG indices may affect the structure of specific brain regions, potentially influencing cognitive function. These findings offer fresh perspectives on the link between modified TyG indices and dementia risk, underscoring the vital role of metabolic health in preserving brain structure and cognitive function.

## Data Availability

The original contributions presented in the study are included in the article/Supplementary material, further inquiries can be directed to the corresponding author/s.
