# Supplementary material for: Associations of modified triglyceride-glucose indices with risks of dementia subtypes and brain structure: a prospective cohort study
Source: Front Neurol. 2026 Feb 26;17:1750736. doi: 10.3389/fneur.2026.1750736 (PMC12979095; doi:10.3389/fneur.2026.1750736)
Supplement: Supplementary file 1 [file Table_1.DOCX]

**Supplementary Figure 1** Association of the TyG index and dementia risk using a multivariable-adjusted restricted cubic spines model.


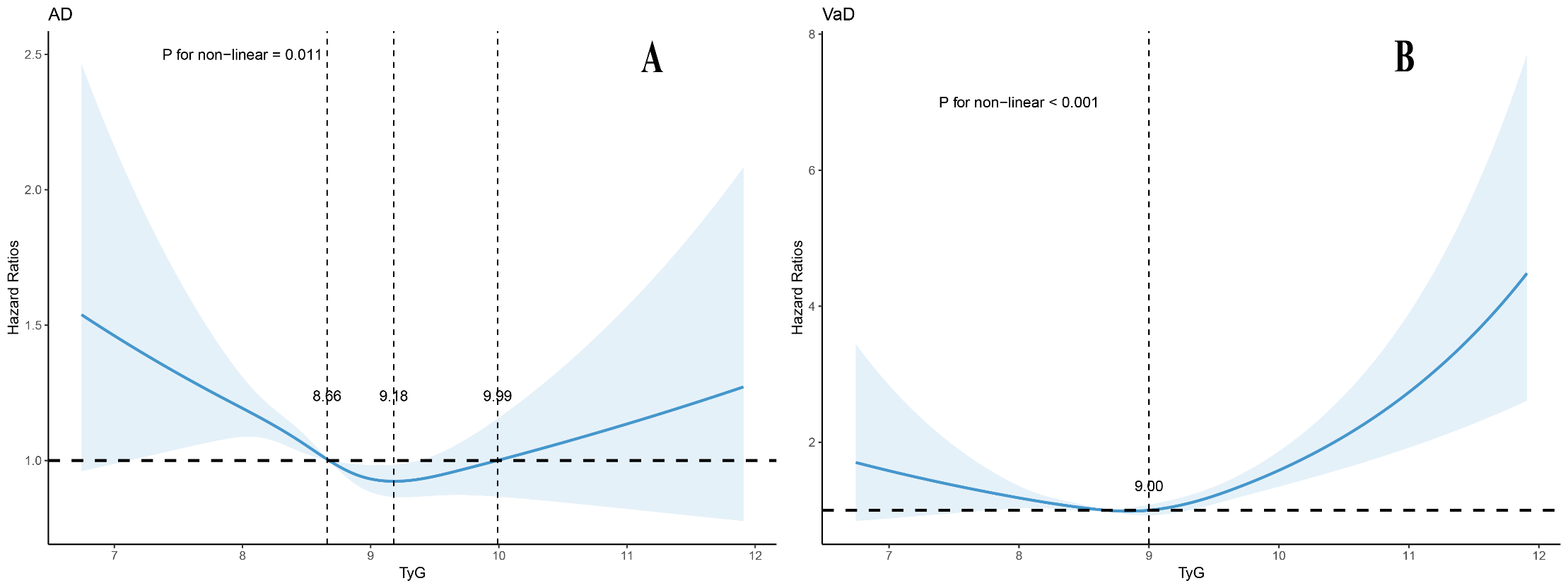


Restricted cubic spline analysis has four knots at the 5th, 35th, 65th, and 95th percentiles of the TyG index. The model adjusted for age, gender, ethnicity, education, and APOE genotype, Townsend deprivation index (TDI), smoking status, drinking status, household income, dietary habits, and sleep patterns, the history of hypertension, hyperlipidemia, diabetes, atrial fibrillation, stroke, the use of glucose-lowering and lipid-lowering medications.

A for participants with AD; B for participants withVaD; TyG, triglyceride-glucose index; AD, Alzheimer’s disease; VaD, vascular dementia.

**Supplementary Table 1** The HR (95% CI) of dementia risk after additional adjustment for relevant brain structural measures.

| **Categories** | **Model 1** | | | **Model 2** | | | **Model 3** | | |
| --- | --- | --- | --- | --- | --- | --- | --- | --- | --- |
|  | **HR (95 % CI)** | **P value** | **Adjusted P** | **HR (95 % CI)** | **P value** | **Adjusted P** | **HR (95 % CI)** | **P value** | **Adjusted P** |
| **AD** |  |  |  |  |  |  |  |  |  |
| TyG-BMI |  |  |  |  |  |  |  |  |  |
| Continuous variable per unit | 0.999 (0.9982, 0.9999) | 0.022* | 0.078 | 0.9985 (0.9976, 0.9993) | <0.001* | 0.002* | 0.9942 (0.9866, 1.0018) | 0.137 | 0.674 |
| Sextile |  |  |  |  |  |  |  |  |  |
| Q1 | 1.42 (1.24, 1.62) | <0.001* | <0.001* | 1.46 (1.27, 1.66) | <0.001* | <0.001* | 1.45 (0.55, 3.8) | 0.448 | 0.678 |
| Q2 | 1.1 (0.97, 1.26) | 0.14 | 0.229 | 1.14 (1, 1.3) | 0.052 | 0.094 | 1.43 (0.56, 3.66) | 0.452 | 0.678 |
| Q3 | 1.13 (1, 1.28) | 0.055 | 0.121 | 1.16 (1.03, 1.32) | 0.018* | 0.047* | 0.52 (0.16, 1.66) | 0.272 | 0.674 |
| Q4 | 1.11 (0.98, 1.26) | 0.09 | 0.162 | 1.13 (1, 1.28) | 0.051 | 0.094 | 0.79 (0.28, 2.29) | 0.67 | 0.919 |
| Q5 | Reference |  |  | Reference |  |  | Reference |  |  |
| Q6 | 1.17 (1.03, 1.32) | 0.017* | 0.078 | 1.12 (0.98, 1.27) | 0.089 | 0.134 | 1.2 (0.4, 3.6) | 0.742 | 0.919 |
| TyG-WC |  |  |  |  |  |  |  |  |  |
| Continuous variable per unit | 0.9999 (0.9996, 1.0002) | 0.431 | 0.518 | 0.9997 (0.9994, 1) | 0.044* | 0.094 | 0.9987 (0.9961, 1.0013) | 0.337 | 0.674 |
| Sextile |  |  |  |  |  |  |  |  |  |
| Q1 | 1.17 (1.01, 1.35) | 0.035* | 0.09 | 1.22 (1.05, 1.41) | 0.008* | 0.024* | 1.46 (0.55, 3.9) | 0.446 | 0.678 |
| Q2 | 1.16 (1.02, 1.32) | 0.026* | 0.078 | 1.19 (1.05, 1.36) | 0.008* | 0.024* | 0.86 (0.32, 2.32) | 0.766 | 0.919 |
| Q3 | 1.04 (0.91, 1.18) | 0.56 | 0.63 | 1.07 (0.94, 1.21) | 0.311 | 0.35 | 0.9 (0.35, 2.3) | 0.818 | 0.92 |
| Q4 | 1.05 (0.93, 1.19) | 0.418 | 0.518 | 1.07 (0.95, 1.21) | 0.278 | 0.333 | 0.52 (0.17, 1.57) | 0.248 | 0.674 |
| Q5 | Reference |  |  | Reference |  |  | Reference |  |  |
| Q6 | 1.12 (0.99, 1.27) | 0.06 | 0.121 | 1.09 (0.96, 1.23) | 0.184 | 0.237 | 0.94 (0.33, 2.65) | 0.906 | 0.959 |
| **VaD** |  |  |  |  |  |  |  |  |  |
| TyG-BMI |  |  |  |  |  |  |  |  |  |
| Continuous variable per unit | 1.0047 (1.0036, 1.0057) | <0.001* | <0.001* | 1.0038 (1.0027, 1.0049) | <0.001* | <0.001* | 1.0097 (1.0003, 1.0193) | 0.043* | 0.26 |
| Sextile |  |  |  |  |  |  |  |  |  |
| Q1 | Reference |  |  | Reference |  |  | Reference |  |  |
| Q2 | 0.83 (0.66, 1.05) | 0.116 | 0.262 | 0.85 (0.68, 1.07) | 0.168 | 0.324 | 0.2 (0.03, 1.54) | 0.123 | 0.317 |
| Q3 | 1.03 (0.83, 1.27) | 0.781 | 0.827 | 1.05 (0.85, 1.3) | 0.652 | 0.691 | 0.38 (0.09, 1.65) | 0.196 | 0.392 |
| Q4 | 0.95 (0.77, 1.17) | 0.606 | 0.728 | 0.94 (0.76, 1.17) | 0.6 | 0.675 | 0.66 (0.19, 2.28) | 0.51 | 0.664 |
| Q5 | 1.09 (0.89, 1.34) | 0.405 | 0.521 | 1.06 (0.86, 1.31) | 0.57 | 0.675 | 0.2 (0.03, 1.48) | 0.115 | 0.317 |
| Q6 | 1.59 (1.3, 1.94) | <0.001* | <0.001* | 1.46 (1.19, 1.78) | <0.001* | 0.001* | 2.23 (0.88, 5.66) | 0.09 | 0.317 |
| TyG-WC |  |  |  |  |  |  |  |  |  |
| Continuous variable per unit | 1.0018 (1.0014, 1.0022) | <0.001* | <0.001* | 1.0014 (1.001, 1.0018) | <0.001* | <0.001* | 1.0038 (1.0002, 1.0073) | 0.036* | 0.26 |
| Sextile |  |  |  |  |  |  |  |  |  |
| Q1 | Reference |  |  | Reference |  |  | Reference |  |  |
| Q2 | 1.15 (0.9, 1.46) | 0.273 | 0.446 | 1.12 (0.88, 1.43) | 0.345 | 0.564 | 1.16 (0.34, 4) | 0.817 | 0.919 |
| Q3 | 1.12 (0.88, 1.42) | 0.349 | 0.506 | 1.09 (0.86, 1.39) | 0.481 | 0.675 | 0.25 (0.03, 1.9) | 0.181 | 0.392 |
| Q4 | 1.28 (1.01, 1.62) | 0.039* | 0.101 | 1.23 (0.97, 1.55) | 0.089 | 0.266 | 0.93 (0.27, 3.22) | 0.912 | 0.945 |
| Q5 | 1.16 (0.91, 1.47) | 0.225 | 0.414 | 1.08 (0.85, 1.37) | 0.542 | 0.675 | 0.3 (0.04, 2.26) | 0.243 | 0.438 |
| Q6 | 1.86 (1.48, 2.34) | <0.001* | <0.001* | 1.63 (1.3, 2.06) | <0.001* | <0.001* | 2.83 (1.12, 7.17) | 0.028* | 0.26 |

* indicates statistical significance.

Model 1 was adjusted for age, gender, ethnicity, education, and APOE genotype.

Model 2 was further adjusted for Townsend deprivation index (TDI), smoking status, drinking status, household income, dietary habits, and sleep patterns.

Model 3 was additionally adjusted for a history of hypertension, hyperlipidemia, diabetes, atrial fibrillation, and stroke, as well as the use of glucose-lowering medications and lipid-lowering medications. Hippocampal volume was additionally adjusted for analyses of Alzheimer’s disease, and white matter hyperintensity (WMH) volume was additionally adjusted for analyses of vascular dementia.

TyG, triglyceride-glucose index; BMI, body mass index; WC, waist circumference; TyG-BMI, TyG combining with body mass index; TyG-WC, TyG combining with waist circumference; AD, Alzheimer’s disease; VaD, vascular dementia; HR, hazard ratios; CI, confidence interval.

**Supplementary Table 2 Subgroup analyses of the association between modified TyG indices and dementia.**

|  |  | **TyG-BMI** | | | **TyG-WC** | | |
| --- | --- | --- | --- | --- | --- | --- | --- |
| **Variables** | **Subgroup** | **HR (95% CI)** | **P value** | **P for interaction** | **HR (95% CI)** | **P value** | **P for interaction** |
| **AD** |  |  |  |  |  |  |  |
| **Age** |  |  |  | **0.003*** |  |  | **<0.001*** |
|  | **≥ 65** | 0.9976 (0.9965, 0.9987) | <0.001 |  | 0.9994 (0.999, 0.9998) | 0.002 |  |
|  | **<65** | 0.9988(0.9975, 0.9997) | 0.057 |  | 1.0001(0.9996, 1.0005) | 0.685 |  |
| **Sex** |  |  |  | 0.47 |  |  | 0.207 |
|  | **Female** | 0.9982(0.9971, 0.9993) | 0.001 |  | 0.9998(0.9994, 1.0002) | 0.421 |  |
|  | **Male** | 0.9982(0.9969, 0.9995) | 0.005 |  | 0.9996(0.9992, 1.0001) | 0.089 |  |
| **Ethnicity** |  |  |  | 0.117 |  |  | 0.976 |
|  | **White** | 0.9984(0.9975, 0.9992) | <0.001 |  | 0.9997(0.9994, 1.0001) | 0.103 |  |
|  | **Non-white** | 0.9948(0.9906, 0.9989) | 0.014 |  | 0.9995(0.998, 1.0009) | 0.471 |  |
| **APOE** |  |  |  | **0.003*** |  |  | **<0.001*** |
|  | **ε4 Carrier** | 0.9993(0.998, 1.0007) | 0.327 |  | 1.0003(0.9998, 1.0007) | 0.262 |  |
|  | **ε4 Non-carrier** | 0.9975(0.9965, 0.9986) | <0.001 |  | 0.9994(0.999, 0.9998) | 0.003 |  |
| **Hypertension** |  |  |  | **0.019*** |  |  | 0.106 |
|  | **Yes** | 0.9968(0.9954, 0.9983) | <0.001 |  | 0.9995(0.9989, 1) | 0.045 |  |
|  | **No** | 0.9988(0.9978, 0.9998) | 0.022 |  | 0.9998(0.9995, 1.0002) | 0.373 |  |
| **Hyperlipidemia** |  |  |  | **0.019*** |  |  | 0.141 |
|  | **Yes** | 0.9896(0.9835, 0.9958) | 0.001 |  | 0.9968(0.9948, 0.9989) | 0.003 |  |
|  | **No** | 0.9984(0.9975, 0.9992) | <0.001 |  | 0.9998(0.9995, 1.0001) | 0.193 |  |
| **Diabetes** |  |  |  | 0.179 |  |  | **0.007*** |
|  | **Yes** | 0.9952(0.9907, 0.9997) | 0.036 |  | 0.9981(0.9965, 0.9997) | 0.02 |  |
|  | **No** | 0.9983(0.9974, 0.9991) | <0.001 |  | 0.9998(0.9995, 1.0001) | 0.164 |  |
| **Fasting duration** |  |  |  | 0.15 |  |  | 0.448 |
|  | **≥8 hours** | 0.9957(0.9918, 0.9997) | 0.034 |  | 0.9994(0.998, 1.0008) | 0.383 |  |
|  | **<8 hours** | 0.9983(0.9975, 0.9992) | <0.001 |  | 0.9998(0.9995, 1.0001) | 0.122 |  |
|  |  |  |  |  |  |  |  |
| **VaD** |  |  |  |  |  |  |  |
| **Age** |  |  |  | **<0.035*** |  |  | **<0.001*** |
|  | **≥ 65** | 1.0025 (1.001, 1.0039) | <0.001 |  | 1.0009 (1.0004, 1.0015) | <0.001 |  |
|  | **<65** | 1.0027(1.001, 1.0043) | 0.002 |  | 1.0013 (1.0007, 1.002) | <0.001 |  |
| **Sex** |  |  |  | 0.555 |  |  | 0.729 |
|  | **Female** | 1.0024(1.0008, 1.0039) | 0.004 |  | 1.0011 (1.0005, 1.0017) | <0.001 |  |
|  | **Male** | 1.0029(1.0014, 1.0044) | <0.001 |  | 1.0012 (1.0007, 1.0018) | <0.001 |  |
| **Ethnicity** |  |  |  | 0.971 |  |  | 0.856 |
|  | **White** | 1.0027(1.0016, 1.0038) | <0.001 |  | 1.0012 (1.0008, 1.0016) | <0.001 |  |
|  | **Non-white** | 1.002(0.997, 1.0072) | 0.431 |  | 1.0007 (0.9988, 1.0026) | 0.452 |  |
| **APOE** |  |  |  | **0.005*** |  |  | **<0.001*** |
|  | **ε4 Carrier** | 1.0038(1.0023, 1.0053) | <0.001 |  | 1.0017 (1.0012, 1.0023) | <0.001 |  |
|  | **ε4 Non-carrier** | 1.0015(0.9999, 1.0031) | 0.062 |  | 1.0006 (1, 1.0012) | 0.053 |  |
| **Hypertension** |  |  |  | 0.643 |  |  | 0.5 |
|  | **Yes** | 1.0025(1.0007, 1.0042) | 0.005 |  | 1.0012 (1.0006, 1.0019) | <0.001 |  |
|  | **No** | 1.0026(1.0012, 1.004) | <0.001 |  | 1.0011 (1.0006, 1.0016) | <0.001 |  |
| **Hyperlipidemia** |  |  |  | 0.638 |  |  | 0.486 |
|  | **Yes** | 1.0019(0.9938, 1.0102) | 0.642 |  | 1.0009 (0.998, 1.0038) | 0.539 |  |
|  | **No** | 1.0027(1.0016, 1.0038) | <0.001 |  | 1.0012 (1.0008, 1.0016) | <0.001 |  |
| **Diabetes** |  |  |  | 0.879 |  |  | 0.944 |
|  | **Yes** | 1.0037(0.9987, 1.0087) | 0.143 |  | 1.0007 (0.9989, 1.0026) | 0.432 |  |
|  | **No** | 1.0026(1.0015, 1.0038) | <0.001 |  | 1.0012 (1.0008, 1.0016) | <0.001 |  |
| **Fasting duration** |  |  |  | 0.947 |  |  | 0.806 |
|  | **≥8 hours** | 1.0029(0.9981, 1.0077) | 0.236 |  | 1.0017 (0.9999, 1.0035) | 0.063 |  |
|  | **<8 hours** | 1.0027(1.0015, 1.0038) | <0.001 |  | 1.0011 (1.0007, 1.0016) | <0.001 |  |

* indicates statistical significance.

Model adjusted for age, gender, ethnicity, education, and APOE genotype, Townsend deprivation index (TDI), smoking status, drinking status, household income, dietary habits, and sleep patterns, the history of hypertension, hyperlipidemia, diabetes, atrial fibrillation, stroke, the use of glucose-lowering and lipid-lowering medications.

n: number of participants; HR, hazard ratios; CI, confidence interval; TyG, triglyceride-glucose index; BMI, body mass index; WC, waist circumference; TyG-BMI, TyG combining with body mass index; TyG-WC, TyG combining with waist circumference.

**Supplementary Table 3** The HR (95% CI) of dementia risk after excluding participants who developed dementia within the first two years.

| **Categories** | **Model 1** | | | **Model 2** | | | **Model 3** | | |
| --- | --- | --- | --- | --- | --- | --- | --- | --- | --- |
|  | **HR (95 % CI)** | **P value** | **Adjusted P** | **HR (95 % CI)** | **P value** | **Adjusted P** | **HR (95 % CI)** | **P value** | **Adjusted P** |
| **AD** |  |  |  |  |  |  |  |  |  |
| TyG-BMI |  |  |  |  |  |  |  |  |  |
| Continuous variable per unit | 0.9991 (0.9983, 1) | 0.041* | 0.112 | 0.9986 (0.9977, 0.9994) | 0.001* | 0.005* | 0.9984 (0.9975, 0.9993) | <0.001* | 0.002* |
| Sextile |  |  |  |  |  |  |  |  |  |
| Q1 | 1.41 (1.23, 1.61) | <0.001* | <0.001* | 1.44 (1.26, 1.65) | 0.033* | 0.075 | 1.46 (1.27, 1.67) | <0.001* | <0.001* |
| Q2 | 1.09 (0.95, 1.24) | 0.224 | 0.366 | 1.12 (0.98, 1.28) | <0.001* | 0.002* | 1.13 (0.99, 1.29) | 0.065 | 0.117 |
| Q3 | 1.11 (0.98, 1.26) | 0.108 | 0.217 | 1.14 (1, 1.29) | 0.149 | 0.206 | 1.15 (1.01, 1.31) | 0.029* | 0.075 |
| Q4 | 1.1 (0.97, 1.25) | 0.124 | 0.222 | 1.12 (0.99, 1.27) | 0.14 | 0.206 | 1.13 (1, 1.28) | 0.059 | 0.117 |
| Q5 | Reference |  |  | Reference |  |  | Reference |  |  |
| Q6 | 1.16 (1.02, 1.32) | 0.021 | 0.112 | 1.11 (0.98, 1.26) | 0.512 | 0.542 | 1.1 (0.97, 1.25) | 0.143 | 0.206 |
| TyG-WC |  |  |  |  |  |  |  |  |  |
| Continuous variable per unit | 0.9999 (0.9996, 1.0002) | 0.592 | 0.701 | 0.9997 (0.9994, 0.9999) | <0.001* | 0.002* | 0.9997 (0.9994, 0.9998) | 0.033* | 0.045* |
| Sextile |  |  |  |  |  |  |  |  |  |
| Q1 | 1.16 (1, 1.34) | 0.047* | 0.112 | 1.21 (1.04, 1.4) | 0.839 | 0.839 | 1.22 (1.05, 1.41) | 0.009* | 0.031* |
| Q2 | 1.14 (1, 1.3) | 0.047* | 0.112 | 1.18 (1.03, 1.34) | <0.001* | <0.001* | 1.18 (1.04, 1.35) | 0.012* | 0.037* |
| Q3 | 1.03 (0.91, 1.17) | 0.662 | 0.701 | 1.06 (0.93, 1.2) | 0.065 | 0.117 | 1.06 (0.94, 1.21) | 0.34 | 0.382 |
| Q4 | 1.05 (0.93, 1.19) | 0.409 | 0.526 | 1.07 (0.95, 1.21) | 0.029* | 0.075 | 1.08 (0.95, 1.22) | 0.243 | 0.291 |
| Q5 | Reference |  |  | Reference |  |  | Reference |  |  |
| Q6 | 1.13 (1, 1.28) | 0.05 | 0.112 | 1.09 (0.97, 1.23) | 0.059 | 0.117 | 1.08 (0.95, 1.22) | 0.242 | 0.291 |
| **VaD** |  |  |  |  |  |  |  |  |  |
| TyG-BMI |  |  |  |  |  |  |  |  |  |
| Continuous variable per unit | 1.0047 (1.0036, 1.0058) | <0.001* | <0.001* | 1.0038 (1.0027, 1.0049) | <0.001* | <0.001* | 1.003 (1.0019, 1.0042) | <0.001* | <0.001* |
| Sextile |  |  |  |  |  |  |  |  |  |
| Q1 | Reference |  |  | Reference |  |  | Reference |  |  |
| Q2 | 0.86 (0.68, 1.08) | 0.197 | 0.407 | 0.88 (0.7, 1.11) | 0.273 | 0.491 | 0.87 (0.69, 1.1) | 0.249 | 0.449 |
| Q3 | 1.04 (0.84, 1.29) | 0.701 | 0.788 | 1.06 (0.86, 1.32) | 0.577 | 0.655 | 1.04 (0.84, 1.28) | 0.739 | 0.89 |
| Q4 | 0.96 (0.77, 1.19) | 0.688 | 0.788 | 0.96 (0.77, 1.19) | 0.685 | 0.725 | 0.92 (0.74, 1.14) | 0.439 | 0.659 |
| Q5 | 1.12 (0.91, 1.38) | 0.299 | 0.449 | 1.09 (0.88, 1.34) | 0.43 | 0.646 | 1.01 (0.82, 1.25) | 0.901 | 0.976 |
| Q6 | 1.62 (1.32, 1.98) | <0.001* | <0.001* | 1.49 (1.21, 1.82) | <0.001* | 0.001* | 1.34 (1.09, 1.65) | 0.005* | 0.024* |
| TyG-WC |  |  |  |  |  |  |  |  |  |
| Continuous variable per unit | 1.0018 (1.0014, 1.0022) | <0.001* | <0.001* | 1.0014 (1.001, 1.0018) | <0.001* | <0.001* | 1.0012 (1.0007, 1.0016) | <0.001* | <0.001* |
| Sextile |  |  |  |  |  |  |  |  |  |
| Q1 | Reference |  |  | Reference |  |  | Reference |  |  |
| Q2 | 1.16 (0.91, 1.48) | 0.241 | 0.407 | 1.14 (0.89, 1.45) | 0.305 | 0.5 | 1.11 (0.87, 1.42) | 0.389 | 0.636 |
| Q3 | 1.11 (0.87, 1.41) | 0.404 | 0.559 | 1.08 (0.85, 1.37) | 0.542 | 0.655 | 1.04 (0.82, 1.33) | 0.742 | 0.89 |
| Q4 | 1.3 (1.02, 1.65) | 0.031* | 0.079 | 1.24 (0.98, 1.58) | 0.07 | 0.21 | 1.18 (0.93, 1.5) | 0.171 | 0.444 |
| Q5 | 1.15 (0.9, 1.47) | 0.249 | 0.407 | 1.07 (0.84, 1.37) | 0.572 | 0.655 | 1 (0.78, 1.27) | 0.976 | 0.976 |
| Q6 | 1.88 (1.49, 2.37) | <0.001* | <0.001* | 1.65 (1.31, 2.09) | <0.001* | <0.001* | 1.47 (1.16, 1.86) | 0.001* | 0.009* |

* indicates statistical significance.

Model 1 was adjusted for age, gender, ethnicity, education, and APOE genotype.

Model 2 was further adjusted for Townsend deprivation index (TDI), smoking status, drinking status, household income, dietary habits, and sleep patterns.

Model 3 was additionally adjusted for a history of hypertension, hyperlipidemia, diabetes, atrial fibrillation, stroke, and the use of glucose-lowering and lipid-lowering medications.

TyG, triglyceride-glucose index; BMI, body mass index; WC, waist circumference; TyG-BMI, TyG combining with body mass index; TyG-WC, TyG combining with waist circumference; AD, Alzheimer’s disease; VaD, vascular dementia; HR, hazard ratios; CI, confidence interval.

**Supplementary Table 4** The HR (95% CI) of dementia according to the TyG index in the three Models.

| **Categories** | **Model 1** | |  | **Model 2** | |  | **Model 3** | |  |
| --- | --- | --- | --- | --- | --- | --- | --- | --- | --- |
|  | **HR (95 % CI)** | **P value** | **Adjusted P**  **(FDR)** | **HR (95 % CI)** | **P value** | **Adjusted P**  **(FDR)** | **HR (95 % CI)** | **P value** | **Adjusted P (FDR)** |
| **AD** |  |  |  |  |  |  |  |  |  |
| TyG |  |  |  |  |  |  |  |  |  |
| Continuous variable per unit | 0.91 (0.85, 0.98) | 0.009* | 0.051 | 0.89 (0.83, 0.95) | 0.001* | 0.004* | 0.88 (0.82, 0.94) | < 0.001* | 0.001* |
| Sextile |  |  |  |  |  |  |  |  |  |
| Q1 | 1.27 (1.11, 1.45) | < 0.001* | 0.004* | 1.3 (1.14, 1.49) | < 0.001* | 0.001* | 1.3 (1.14, 1.49) | < 0.001* | 0.001* |
| Q2 | 1.09 (0.96, 1.24) | 0.161 | 0.242 | 1.12 (0.98, 1.27) | 0.087 | 0.134 | 1.12 (0.99, 1.27) | 0.080 | 0.131 |
| Q3 | 1.08 (0.95, 1.22) | 0.222 | 0.307 | 1.1 (0.97, 1.24) | 0.130 | 0.180 | 1.1 (0.98, 1.25) | 0.114 | 0.171 |
| Q4 | 1.03 (0.91, 1.16) | 0.666 | 0.705 | 1.04 (0.92, 1.18) | 0.513 | 0.543 | 1.04 (0.92, 1.18) | 0.524 | 0.555 |
| Q5 | Reference |  |  | Reference |  |  | Reference |  |  |
| Q6 | 1.01 (0.89, 1.14) | 0.853 | 0.853 | 1 (0.88, 1.13) | 0.962 | 0.962 | 1.02 (0.87, 1.11) | 0.772 | 0.772 |
| **VaD** |  |  |  |  |  |  |  |  |  |
| TyG |  |  |  |  |  |  |  |  |  |
| Continuous variable per unit | 1.24 (1.13, 1.37) | < 0.001* | < 0.001* | 1.18 (1.07, 1.30) | 0.001* | 0.002* | 1.14 (1.03, 1.26) | 0.008* | 0.029* |
| Sextile |  |  |  |  |  |  |  |  |  |
| Q1 | Reference |  |  | Reference |  |  | Reference |  |  |
| Q2 | 0.99 (0.8, 1.22) | 0.937 | 0.937 | 0.98 (0.79, 1.21) | 0.851 | 0.851 | 0.99 (0.8, 1.22) | 0.907 | 0.980 |
| Q3 | 0.88 (0.71, 1.09) | 0.230 | 0.414 | 0.86 (0.7, 1.07) | 0.173 | 0.324 | 0.87 (0.7, 1.07) | 0.195 | 0.406 |
| Q4 | 0.96 (0.78, 1.18) | 0.691 | 0.777 | 0.93 (0.76, 1.15) | 0.516 | 0.675 | 0.93 (0.76, 1.14) | 0.473 | 0.655 |
| Q5 | 0.91 (0.74, 1.12) | 0.366 | 0.506 | 0.87 (0.71, 1.07) | 0.180 | 0.324 | 0.86 (0.7, 1.06) | 0.156 | 0.400 |
| Q6 | 1.27 (1.04, 1.54) | 0.017* | 0.051 | 1.17 (0.96, 1.42) | 0.110 | 0.282 | 1.13 (0.93, 1.37) | 0.225 | 0.406 |

* indicates statistical significance.

Model 1 was adjusted for age, gender, ethnicity, education, and APOE genotype.

Model 2 was further adjusted for Townsend deprivation index (TDI), smoking status, drinking status, household income, dietary habits, and sleep patterns.

Model 3 was additionally adjusted for a history of hypertension, hyperlipidemia, diabetes, atrial fibrillation, stroke, and the use of antidiabetic and lipid-lowering medications.

TyG, triglyceride-glucose index; AD, Alzheimer’s disease; VaD, vascular dementia; HR, hazard ratios; CI, confidence interval.
